# Integrating KPFM Characterisation, COMSOL Multiphysics Simulation and Physics-Informed cVAE for Multi-Polymer Triboelectric Nanogenerator Optimisation

**DOI:** 10.3390/ma19091790

**Published:** 2026-04-28

**Authors:** T. Pavan Rahul, P. S. Rama Sreekanth

**Affiliations:** School of Mechanical Engineering, VIT-AP University, Amravati 522237, Andhra Pradesh, India

**Keywords:** triboelectric nanogenerators (TENGs), COMSOL Multiphysics, atomic force microscopy, conditional variational autoencoder (cVAE)

## Abstract

Triboelectric nanogenerators (TENGs) offer a promising route for self-powered microscale energy harvesting, yet their design optimisation remains empirically challenging due to the complex interplay of material surface physics, device geometry and operating mode. In this work, we present an integrated framework that combines atomic force microscopy (AFM) characterisation, COMSOL Multiphysics 6.0 finite element simulation and physics-informed conditional variational autoencoder (cVAE) to predict and optimise TENG output performance. Four polymer dielectric materials, HDPE, LDPE, TPU, and PMMA, were characterised via Kelvin Probe Force microscopy (KPFM) for work function, surface potential and surface roughness. Surface charge density was calculated from measured KPFM potential using the parallel plate capacitor model and used as a boundary condition in COMSOL Multiphysics simulations for contact-separation and lateral sliding TENG mode for dielectric film thicknesses of 50 µm and 100 µm. The simulated open circuit voltage (Voc) and short circuit charge (Qsc) across gap distances up to 150 mm formed the training dataset for a cVAE model with eight physicochemical condition features. The trained model demonstrated strong reconstruction accuracy (R^2^ ≥ 0.94) and enables generative prediction across unseen design spaces. Results reveal that the LDPE/TPU pair at 50 µm thickness consistently achieves the highest electric outputs in both modes, and the sliding mode yields 25–30% higher voltages than the contact separation mode across all material pairs. This study provides a transferable data-efficient methodology for accelerating TENG material and geometry optimisation.

## 1. Introduction

The Internet of Things and wearable technology have exploded onto the technological scene and completely changed the way we power our distributed sensor networks. Devices such as those that monitor structural health throughout smart cities or track physiological activity in real time in the healthcare setting form the networked “nervous system” for our modern society. Today, however, as the limits (both in terms of availability and environmental impact) of fossil fuels become increasingly evident, the scientific community has shifted to developing sustainable and green energy alternatives [1,2]. Triboelectric nanogenerators (TENGs), which exploit contact electrification and electric induction to convert mechanical motion into electrical energy, have emerged as compelling candidates for self-powered systems owing to their low cost, material versatility and high-power density at low operational frequencies [3,4]. The full potential of TENGs will be realised through their ability to provide power, as well as to act as self-powered sensors [5,6]. The TENG generates an electrical signal that is directly proportional to the mechanical stimulus applied (force, frequency, and displacement) [7]. The operation of the TENG is based on and takes advantage of dual mechanisms: contact electrification (CE) and electrostatic induction (EI). When a dissimilar material contacts another (i.e., materials that have different electron affinities or triboelectric effects), the two surfaces will exchange surface charge during the act of contact. As the two surfaces separate or move relative to each other, a potential difference develops at the interface, which causes electron flow through the external circuit, thereby creating a balance to the potential. According to initial tests, the output performance of the TENG, typically expressed in terms of open-circuit voltage (Voc), short-circuit current (Isc), and transferred charge (QT), is dependent on the properties and geometry of both dielectric materials and TENG topology [8,9]. Of the many factors that affect TENG output performance, the surface charge density (σ) is the key parameter. Although the principles of physics that govern TENGs (CE and EI) have been established, the greatest barrier to achieving commercial volumes and high-power density from TENGs remains the low amount of charge that can be generated and retained on the dielectric surface. According to the TENG, electrical output scales as a function of the square of the surface charge density (P ∝ σ2) [10,11]. Thus, doubling a given charge density would produce a fourfold increase in power output. This means that surface charge densities (σ), which are the primary drivers of TENG output, cannot be treated as a constant material property (e.g., density or Young’s Modulus) but must be viewed instead as highly dynamic surface properties.

TENGs operate through two primary mechanisms: contact separation mode [12], in which two dielectric surfaces repeatedly make and break contact perpendicularly to the interface, and lateral sliding mode, in which surfaces translate parallel to the interface. This electrical output is characterised by open circuit voltage and short circuit charge transfer, which depends on a constellation of material and geometric parameters, including surface charge density, dielectric constant, film thickness and inter-electrode gap distance [13,14,15]. Material selection for TENG dielectric layers conventionally relies on the triboelectric series, a qualitative ranking of materials by their tendency to donate or accept surface charge. However, this heuristic fails to capture quantitative differences in surface potential, work function and nanoscale morphology that critically govern actual charge transfer magnitude [16]. The atomic force microscopy (AFM) technique, particularly Kelvin Probe Force microscopy (KPFM) provides direct nanometre-resolved measurement of contact potential difference and work function, enabling physics-based prediction of interfacial charge exchange [17].

In recent years, machine learning and deep learning, with its powerful automation capabilities and adaptive learning features, has found widespread application in the field of triboelectric nanogenerators [18]. For example, Okbar et al. numerically investigated the effect of various material parameters and operating conditions on the voltage, current and power output of TENG. Results show that surface charge density consistently boosts output, while dielectric properties, area, and velocity are most effective at low to medium load resistance. Conversely, separation distance exhibits a load-dependent dual effect, improving performance only at high resistances. These data were leveraged to train a multilayer perceptron (MLP) neural network (9-9-55-3 architecture) providing a robust machine-learning-based simulator for predicting TENG current, voltage and power [19]. Wu et al. proposed an attention-based convolutional neural network (AttCNN) to bridge the reliability gap in TENG electronic components through real-time fault diagnosis. This model integrates an attention mechanism for global signal correlation with a convolutional network for effective feature extraction [20].

The goal of this research is to develop a systematic study of how to optimise the performance of triboelectric nanogenerators (TENGs) by creating an empirical relationship between the nanoscale surface characteristics of the materials used in the construction of TENGs and their performance in operation on a macroscale as predicted by finite element analysis in COMSOL Multiphysics 6.0 software. This study has three objectives: nanoscale experimental characterisation of four candidate polymers (HDPE, LDPE, TPU and PMMA), mathematical modelling of the surface charge density (σ) from the experimental data on electrical potentials determined from testing and modelling of multi-mode, and multi-thickness finite element analysis (FEA) through the COMSOL Multiphysics 6.0 software to compare and optimise TENG performance in contact-separation and contact-sliding modes. HDPE and LDPE are being used as the two polymers that correlate in the same family; therefore, the focus of this investigation has been to determine how density and crystallinity will impact the charge retention ability of these polymers. The third polymer being evaluated is TPU, as a representative of the flexible electronic industry, and PMMA has been selected because of its well-defined position in the triboelectric series, and its surface charge behaviour is reproducible and less sensitive to environmental fluctuations. The contact potential difference (VCPD) and calculated values of work function (Φ) have been used as the theoretical basis for defining the Surface Charge Densities for each of the four polymers. These values will provide an important input for each of the finite element analyses performed with COMSOL Multiphysics software and will be determined by varying the thicknesses of the dielectric materials used on each of the four TENGs in the analysis. However, TENGs have been extensively studied as mechanical energy harvesting devices, with their performance strongly influenced by the selection of triboelectric materials, dielectric thickness, surface characteristics, and operating mode. A primary limitation in existing TENG modelling studies is the treatment of surface charge density. In many reports, the surface charge density is assumed to be a fixed value or estimated from the literature. Although this approach allows parametric studies, it does not establish a direct connection between the intrinsic electrical properties of the triboelectric materials and training of a conditional variational autoencoder (cVAE) on the combined experimental and simulation dataset to enable rapid, uncertainty-aware prediction of TENG performance as a function of material pair, thickness, and gap distance. The framework provides both a practical design tool and a generalisable methodology for physics-informed materials-by-design in energy harvesting applications.

## 2. Materials and Methods

### 2.1. Fabrication of Polymer Dielectric Layers

**Figure 1:**
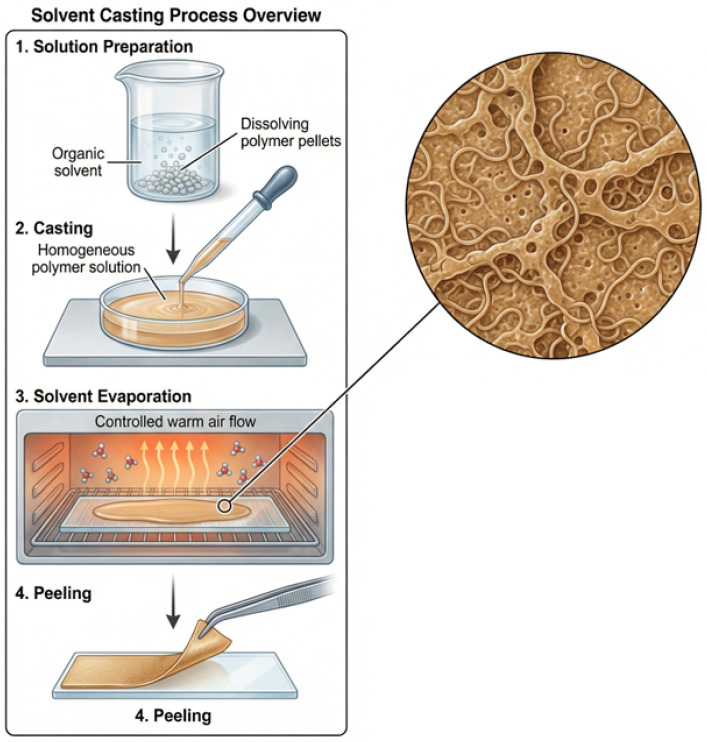
Solution-casted film fabrication process.

As depicted in Figure 1, polymer dielectric layers of HDPE, LDPE, TPU, and PMMA were fabricated using a controlled solvent-casting technique to obtain uniform, defect-minimised thin films suitable for triboelectric nanogenerator (TENG) applications [21]. Solvent casting was selected due to its simplicity, scalability, and ability to preserve the intrinsic physicochemical properties of polymers while enabling precise control over film thickness and surface morphology.

Xylene was selected as a solvent for HDPE and LDPE because of its strong solubility for polyolefin-containing polymers at higher temperatures. Plastic resin pellets were incrementally added to the xylene under constant agitation using magnets. The mixture was stirred at 130–150 °C continuously to completely dissolve the polymer chains. Stirring was performed for about 3 h until a homogeneous solution with a viscosity that had no visible agglomerates was achieved. Maintaining a high processing temperature (increasing the polymer chain disentanglement and uniform distribution of polymer material) is important so that smooth and uniform films can be obtained after the casting process [22,23].

The dissolution process involved pouring polymer solutions into sterile glass Petri dishes on a flat countertop to prevent the formation of thickness variation while also maintaining consistency throughout the production of cast films. Following the casting of the films, they were placed into an oven at a measured temperature for 4–5 h for total control over the temperature throughout the drying phase of the coating process. By subjecting the films to prolonged high temperatures through the use of an oven, the films were completely dry due to the evaporation of the solvent, thus helping with the resin’s solidification through gradual reduction of the solvent from the resin, resulting in reduced levels of residual stress, voids, and surface defects. Once the films were dried, they were allowed to cool to room temperature before being removed by gently pulling them off the petri dish and allowing free-standing dielectric layers to be obtained. In addition, TPU and PMMA were dissolved in N, N-dimethylformamide (DMF), which is an aprotic polar solvent that has an excellent ability to dissolve polar polymers. A polymer was added to DMF, and agitated at low, controlled temperatures of 60–100 °C to avoid temperature-induced degradation of the polymer while having adequate time to dissolve completely into the DMF solution. After approximately 3–4 h of agitation, the DMF and polymer were in a uniform and clear solution. The lower processing temperature is particularly important for TPU due to its segmented molecular structure and sensitivity to excessive thermal exposure. The resulting TPU and PMMA solutions were poured into Petri dishes and dried in a hot air oven for approximately 4 h. Controlled evaporation of DMF allowed the formation of dense, uniform films with minimal porosity.

### 2.2. Atomic Force Microscopy

The topographic properties and surface structure of the polymer films were measured using an Atomic Force Microscope (Park Systems NX20 AFM, Park Systems, Suwon, Republic of Korea) operating in Frequency Modulation (FM-AFM). Before measurements, the polymer films were sectioned into square samples measuring approximately 10 × 10 mm to provide mechanical stability and a valid method for mounting the specimens on the AFM holder. The specimens were attached to metal AFM stubs via conductive adhesive tape to eliminate sample movement and to provide reliable tip/samples interactions while scanning. All of the AFM measurements were performed at ambient lab temperatures. High-resolution surface scans were acquired over a scan area of 10 × 10 µm^2^, which was selected to capture representative nanoscale roughness while avoiding edge effects and macroscopic defects. The system was initially calibrated by a HOPG sample to find out the tip work function. FM-AFM mode was chosen due to its enhanced sensitivity to short-range tip–sample interactions, allowing accurate determination of surface height variations and local mechanical responses without inducing surface damage to the relatively soft polymer films [24].

### 2.3. Derivation of Surface Charge Density from AFM/KPFM Data

To provide a numerical understanding of triboelectric nanogenerator (TENG) performance, it is necessary to make a direct link between electronic properties at the nanoscale level (the surface of the polymer dielectric material) and electrostatic behaviour at a macroscale level. This is accomplished by using Kelvin Probe Force Microscopy (KPFM) in Atomic Force Microscopy (Park Systems NX20, Park Systems) to measure the surface potential of the polymer dielectric, which can then be used to establish an effective surface charge density that is appropriate for use in continuum-level finite element simulations using COMSOL Multiphysics 6.0. This mathematical framework establishes a link between what was observed experimentally at the nanoscale and how it was numerically predicted regarding TENG electrical output.

KPFM measures the contact potential difference (VCPD) between a conductive AFM tip and the surface of the polymer dielectric sample material. When two materials with different work functions (e.g., polymer dielectric A has a lower work function than polymer dielectric B) are brought into proximity to one another, electrons will flow from the material that has a lower work function to the material that has a higher work function until the two materials have equal Fermi levels. This electron redistribution will create an electrostatic potential difference across the two materials, which can be measured by KPFM [25].

The measured contact potential difference is expressed as

V_cpd_ = (ϕ_t_ − ϕ_s_)/e, (1)

where ϕ_t_ and ϕ_s_ represent the work function of the AFM tip and polymer sample, respectively, and e is the elementary charge. In frequency-modulated KPFM (FM-KPFM), an external DC bias is applied to nullify the electrostatic force gradient, and the compensating voltage directly corresponds to VCPD.

An AFM tip was calibrated to obtain absolute work function values of the polymer dielectrics using a reference material that has a known work function, HOPG, to determine the tip’s work function. During the contact electrification of materials with greater differences from the average work function of the counter electrode, charge transfer is more pronounced, leading to a higher surface charge density on the polymer and, thus, an increase in TENG output. Therefore, work function analysis provides an electronic rationale for both the triboelectric polarity and magnitude of charge.

Using KPFM measurements of measured VCPD converted into effective surface charge densities (σ) is how KPFM measurements are integrated into TENG modelling using the compensation model, electrostatic approximation of the polymer dielectric as a uniform charge slab.

Assuming a parallel-plate capacitor configuration, the electric field generated by a surface charge density across a dielectric layer of thickness d and relative permittivity ε_r_ is given by

E = σ/(ε_0_ × ε_r_), (2)

where ε_0_ is the vacuum permittivity, the corresponding electrostatic potential across the dielectric is

V = E × d = σ d/(ε_0_ ε_r_), (3)

By equating the measured surface potential to this electrostatic potential, the effective surface charge density can be expressed as

σ = (ε_0_ ε_r_ V_cpd_)/d, (4)

The resulting σ value represents the surface charge density.

### 2.4. COMSOL Simulation Framework

**Figure 2:**
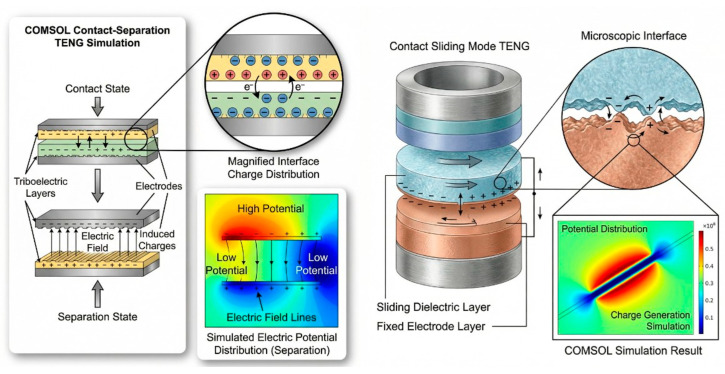
Schematic illustration of COMSOL simulation of contact separation and sliding modes.

Finite element simulations were performed using COMSOL Multiphysics^®^ to investigate the electrical response of polymer-based triboelectric nanogenerators (TENGs) operating in contact-separation (CS) and contact-sliding (SL) modes [26,27]. These simulations were conducted with the electrostatics module inside a 2D electrostatic approximation to accurately model charge transfer at the interface while remaining computationally efficient. The simulation model is shown in Figure 2, with the CS and SL layouts used to simulate these modes.

Two dielectric polymer layers represent the triboelectric pair and are positioned between two metallic electrodes in the simulation domain. Each polymer layer is assigned the appropriate dielectric constant, whereas the electrodes are modelled as equipotential boundaries. Additionally, there is an air domain surrounding the two polymer layers to properly calculate the electric field distribution during separation and sliding. Additionally, electrical insulation boundary conditions will be applied at the outer boundaries of the air domain to prevent artificial charge leakage. One defining feature of this modelling approach is the use of experimental data for surface charge density (σ) as input parameters into the model. Specifically, σ for each polymer interface will be determined from the measured difference in work function and surface potential measured using AFM-KPFM techniques.

For the contact-separation (CS) mode, the separation distance between the two dielectric layers is parametrically varied to simulate periodic contact and release. During contact, opposite surface charges are assumed to fully interact, while, during separation, an increasing air gap leads to the development of a potential difference between the electrodes. The resulting electric field redistribution induces charge flow in the external circuit, allowing extraction of open-circuit voltage (Voc) and short-circuit current (Isc). In the contact-sliding mode, one dielectric layer is translated laterally relative to the other while maintaining constant normal contact. The spatial separation of surface charges along the sliding direction creates a time-varying electric field, inducing alternating current in the external circuit. The 2D model captures charge redistribution along the sliding interface and demonstrates saturation behaviour of the induced potential with increasing sliding displacement. Table 1 shows the material parameters for the COMSOL simulation.

### 2.5. Conditional Variational Autoencoder (cVAE) Model

In this work, we presented a physics-informed conditional variational autoencoder (cVAE) as the core predictive and generative framework for modelling TENG performance. Generative deep learning models allow for probabilistic formulations of the problem of regression and, thus, establishing a principled probabilistic approach to the generation of data through deep learning models provides a framework for learning continuous representations of the latent structure associated with the probabilities of complex data distributions. The Variational Autoencoder (VAE), first proposed by Kingma & Welling (2013), has become the foundation of the development of the methods by which we can learn about the latent structure of a complex data distribution through continuous parameters (e.g., the latent variables of data distributions) [28]. In summary, a VAE consists of two networks: an encoder network that encodes the dataset of observations into a lower-dimensional continuous latent space and reconstructs the observed data from a sample of the posterior distribution (which is parameterised as a multivariate Gaussian distribution with a learned mean and diagonal covariance) using a decoder network and a decoder network, which reconstructs the observations after sampling from the learned posterior distribution. The learning algorithm for training VAEs is to maximise the Evidence Lower Bound (ELBO): L = E[log p(y|z)] − β · KL[q(z|y)∥p(z)], with the first term being the reconstruction likelihood of the sample observed and the second term being the Kullback–Leibler divergence, which biases the posterior to remain close to the standard normal distribution, which helps to establish a smooth and interpolable latent manifold [29]. In contrast to a standard VAE, a Conditional VAE (cVAE) is a specific type of generative deep learning model that incorporates conditional information into the encoding and decoding of the latent representation of the observations. cVAEs provide a probabilistic framework for learning representations of the latent structure in an unsupervised manner and learn a compact, continuous latent representation of the structure of complex data distributions using probabilistic parameters (e.g., the latent variables) across the observed samples. Conditional variational autoencoders (cVAEs), as described by Sohn et al., are an enhancement of standard VAEs via conditioning latent variables on additional auxiliary inputs like physical material properties and/or experimental parameters [30]. In doing so, this transforms the cVAE from being only a generative method to being both an informative and knowledgeable physics-based approach to predicting potential outcomes based on conditions. The cVAE learns not just one mapping between inputs and outputs but rather the entire conditional form of the probability distribution P (y|x), providing the means to quantify predicative uncertainty while also allowing for the exploration of counterfactual scenarios generated through the generation of realistic output for previously unseen material configurations. The encoder now parameterises the conditional posterior Q_ϕ_ (z|y, x), and the decoder learns the conditional generative distribution P_θ_(y|z, x). The model is trained to maximise the conditional ELBO: L = E [log P_θ_(y|z, x)] − β · KL [Q_ϕ_(z|y, x)∥p(z)]. The architecture comprises an encoder network that maps observations to a latent distribution and a decoder network that reconstructs outputs from sampled latent variables conditioned on input features, trained jointly through a combination of reconstruction loss and a Kullback–Leibler (KL) divergence regularisation term. At inference time, multiple independent draws of z~N (0, I) are passed through the decoder conditioned on a target x, yielding a sample ensemble from which the predictive mean and variance are estimated via Monte Carlo integration [31].

#### 2.5.1. Architecture

**Figure 3:**
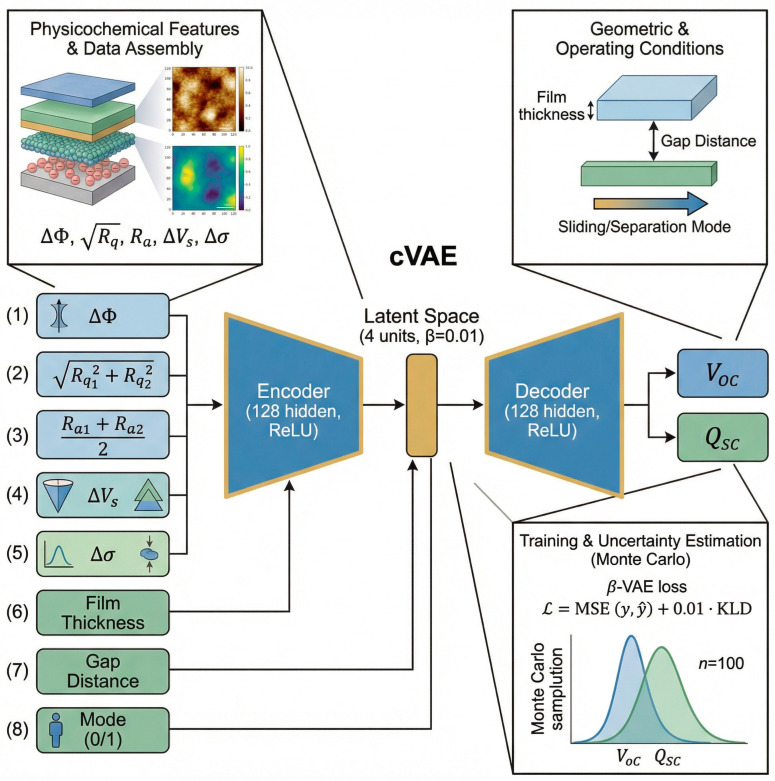
cVAE architecture.

A conditional variational autoencoder (cVAE) [32] was developed to learn the joint distribution of TENG electrical outputs (V_oc_ and Q_sc_) conditioned on eight physicochemical and geometric input features. It comprises two subnetworks: an encoder Q_ϕ_ (z|y,x) and a decoder P_θ_ (y|z, x), both conditioned on the 8-dimensional feature vector x. The conditioning vector comprises: (1) work function difference between the material pair (ΔΦ), (2) combined RMS roughness $\sqrt{R_{q1}^{2}+\;R_{q2}^{2}}$, (3) average arithmetic roughness (Ra_1_ + Ra_2_)/2, (4) KPFM potential difference V_s_, (5) surface charge density difference σ, (6) dielectric film thickness (µm), (7) gap distance (mm), and (8) operating mode (sliding, separation). The model architecture includes a symmetric encoder–decoder structure with fully connected layers (input → 128 hidden units → latent space dimensionality 4) and ReLU activations. The architecture of the cVAE is represented in Figure 3.

#### 2.5.2. Training Procedure

The model was trained using the Adam optimiser (learning rate = 0.005) for 3000 epochs using a β-VAE loss formulation

L = L_RECON_ + β · L^WLD^ = MSE (y, ŷ) + 0.01 · (−½ Σ [1 + log σ^2^ − μ^2^ − σ^2^]), (5)

where the reconstruction loss (MSE) enforces accurate prediction of Voc and Qsc, and the KL-divergence regularisation term (weighted by β = 0.01) enforces a smooth, structured latent space. All input features and output targets were standardised using zero-mean unit-variance scaling before training. The trained model enables Monte Carlo sampling (*n* = 100 latent space draws) to generate uncertainty estimates for any queried design configuration.

#### 2.5.3. Feature Engineering and Data Assembly

Simulation outputs were extracted and paired with corresponding AFM-derived features for each combination of material pairs (4 pairs × 2 modes × 2 thicknesses). Open circuit voltage and short circuit charge values at matched gap positions were assembled into a training dataset of input–output pairs (X, Y), where X ∈ R^8^ and Y ∈ R^2^ for each data point. The final training corpus comprised data points covering gap ranges of 0–9 mm (contact-separation) and 0–150 mm (sliding) at discrete intervals extracted from COMSOL output.

## 3. Results and Discussion

### 3.1. Surface Potential and Work Function Mapping Through KPFM

#### 3.1.1. V_CPD_ Forward/Backwards Stability Analysis

**Figure 4:**
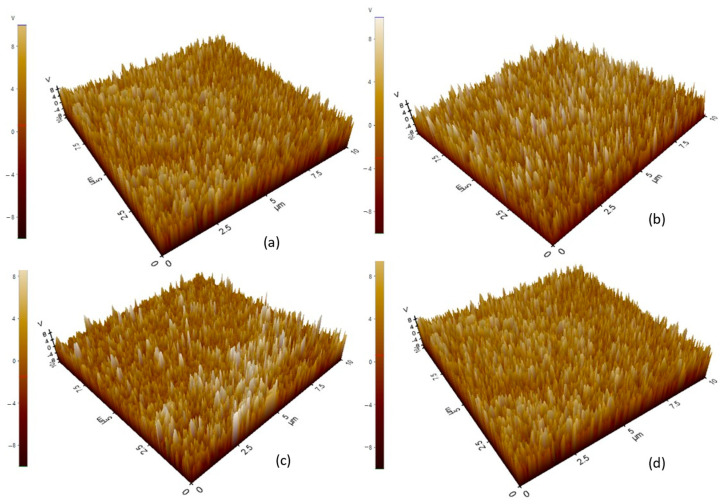
Potential mapping of (**a**) HDPE, (**b**) LDPE, (**c**) PMMA, and (**d**) TPU.

The VCPD is a crucial factor in determining the electronic properties of a surface, as it describes the local work function and charge distribution of a material. It is very important to be able to measure the potential that is available to be used as a charge carrier during contact electrification in a triboelectric nanogenerator (TENG) system. Measuring the surface potential accurately allows you to determine the surface charge density, electron affinity, and the difference in work functions, all of which are critical to the process of charge transfer during contact electrification. Measurements of surface potential reliability can be made with both forward and backward KPFM scans [16]. Discrepancies between the scans may occur due to capacitive coupling between the tip and the surface, hysteresis of the electrostatic forces, lag in the feedback loop, and external noise. To determine if a surface potential is a true measurement of the intrinsic properties of the material, it is important to compare the VCPD values obtained from both the forward and backward scans. The potential mapping of the material is depicted in Figure 4a–d. If a VCPD measurement is stable, the forward and backward scan values will exhibit very little variation from one another, indicating that the electrostatic interaction between the AFM tip and sample surface was stable throughout the measurement process.

High-density polyethene (HDPE) has been tested using a forward voltage charge potential difference (V-CPD), resulting in a forward measurement of −0.590 volts and a reverse measurement of −0.611 volts. This small difference of 0.021 volts indicates a very good reproducibility of the measurements and an insignificant hysteresis effect due to electrostatic forces on the measurements. The V-CPD values obtained for HDPE are negative and consistent with the high work function of HDPE (5.795 eV), meaning that the surface potential of HDPE is lower than the reference tip, confirming it as being electronegative according to the triboelectric charging series. The morphology of the HDPE surface reveals predominantly valleys as seen through atomic force microscopy (AFM) analysis, allowing stable charge placement and minimising any lateral charge migration during the KPFM measurement process. Therefore, HDPE has shown stable, reproducible V-CPD values, which provide confidence in using these values in accurately determining work functions and including these values in triboelectric charging models.

Thermoplastic polyurethane (TPU) exhibited a forward VCPD of 0.690 V and a reverse VCPD of 0.675 V with a difference of 0.015 V. Since TPU exhibited a positive value for both forward and reverse VCPD, it can be inferred that TPU’s VCPD is above that of the reference tip, which is consistent with the relatively low work function of TPU (4.513 eV) and its location on the electropositive end of the triboelectric series. The small difference in forward and back values indicates good repeatability while showing that the measurements had relatively more variation compared to that of HDPE, but both forward and back scans showed a relatively similar level of agreement, which confirms that the measured VCPD values were mainly representative of intrinsic electronic properties of the surface. Thus, the consistency of TPU’s VCPD strongly supports the application of TPU and VCPD data as an indicator of comparative triboelectric polarity.

The forward voltage potential (VCPD) of LDPE was 0.227 V, while the backward VCPD was 0.253 V, resulting in a difference of 0.026 V. This is well within the limits of acceptable measurement stability. LDPE has a negative VCPD; therefore, based upon its work function of 5.43 eV, it is located in the electronegative section of the triboelectric series (although not as negative as HDPE does), indicating that the work function difference created by these two polyethene types is caused by crystallinity, degree of molecular disorder and surface defects. Additionally, the electrostatic attraction established by the AFM tip during scanning may be further strengthened by the height of LDPE being nearly symmetrical and moderately rough. The fact that the backward scan had a larger absolute value than the forward scan could be explained by either charge relaxation or delayed feedback during the retrace scan. The excellent agreement between both scans indicates that LDPE consistently provides an accurate measurement of work function and electron affinity.

Based on the analysis results, PMMA has the highest positive surface potential, with forward and backward VCPD values of 1.191 and 1.190, respectively, with a very small difference of only 0.001 V. The very good agreement between the forward and backwardly acquired VCPD values for PMMA represents the highest measurement stability among the four different materials studied and therefore provides a high degree of certainty that the PMMA surface potential data is both accurate and reproducible. The strong positive VCPD exhibited by PMMA is completely consistent with its lowest measured work function for the studied polymers of 4.012 eV, thus verifying that the surface electrons of PMMA are the weakest bound and that the PMMA surface potential is significantly above the reference tip potential. Therefore, PMMA positions firmly as the most electropositive material on the experimental triboelectric series. The near-perfect forward-to-backwards agreement also suggests that PMMA’s surface charge state remained essentially undisturbed throughout the scanning process, indicative of a highly stable surface electronic configuration, likely facilitated by the immobile, deeply trapped charge states associated with PMMA’s polar ester functional groups.

#### 3.1.2. Work Function Mapping

The work function (Φ) indicates how much energy is needed for an electron to leave the Fermi level of a given material and reach the vacuum level [33]. During triboelectric charging of two materials, electrons will migrate from the lower Φ material to the higher Φ material until they establish an electrostatic equilibrium. Therefore, lower Φ materials will be more likely to contribute electrons and become positively charged, while higher Φ materials will more likely accept electrons and become negatively charged.

HDPE has a Φ of 5.795 eV, which is the highest among all the studied polymers. Therefore, HDPE occupies the most electronegative position in the experimental triboelectric series, meaning it has the strongest tendency to accept electrons when brought into contact with any of the other studied materials. HDPE should develop an extremely strong negative charge from triboelectric seeking and hold that charge for a long time, thus making it ideal for long-term applications of triboelectricity that require mechanical durability. LDPE also has a high electronegativity with a measured Φ of 5.43 eV, which is the next most negative material in the triboelectric series. HDPE and LDPE are chemically similar; however, the difference between the values of their work function shows the extent to which crystallinity, disorder of molecules, and defect states on the surface of a material will influence the surface electronic characteristics of a material, with LDPE expected to take on electrons from TPU or PMMA very easily, as both are lower Φ value materials, resulting in an accumulation of a negative charge; whereas, when placed together with HDPE, due to the small difference of the Φ’s of LDPE and HDPE, very little electron transfer is expected between the two (less than expected due to the lower Φ of LDPE), resulting in a correspondingly lower output of triboelectricity for this pairing.

The polyurethane TPU has a work function (Φ) of 4.513 eV, putting it in an intermediate position on the triboelectric series. Because of its moderate work function, TPU will donate electrons to both HDPE and LDPE when they come into contact with each other, consequently obtaining a positive surface charge with both. The polarity of TPU’s molecular structure enables surface charge redistribution and increases mobility of the surface electrons in TPU; this therefore makes it energetically favourable for TPU to donate electrons. Therefore, TPU functions effectively as a positive triboelectric material when in contact with polymers that have higher Ф such as HDPE and LDPE; because of this, TPU is well-suited to be used as a top layer of the positive electrode in TENG designs.

The poly(methyl methacrylate) PMMA has the lowest experimental work function (Ф = 4.012 eV) of all the polymers tested, which places it at the most positive end of the experimental triboelectric series. PMMA’s low Ф indicates that the surface electrons of the PMMA polymer are the weakest; thus, PMMA is the strongest electron donor of the four materials tested. Therefore, upon contacting each of the other tested polymers (TPU, LDPE, and HDPE), it is anticipated that PMMA will donate electrons readily to other polymers and develop a strong positive surface charge. This behaviour is somewhat contrary to PMMA’s conventional classification in many empirical triboelectric series; however, it is consistent with the measured Φ values and reflects the significant role that surface electronic states, polar ester functional groups, and molecular orientation play in determining actual charge transfer behaviour. The position of PMMA at the positive extreme of this series also suggests that PMMA–HDPE pairings, which exhibit the largest Φ difference (5.795 − 4.012 = 1.783 eV) among all material combinations studied, are expected to yield the highest charge separation and the greatest triboelectric output.

#### 3.1.3. Surface Topography

**Figure 5:**
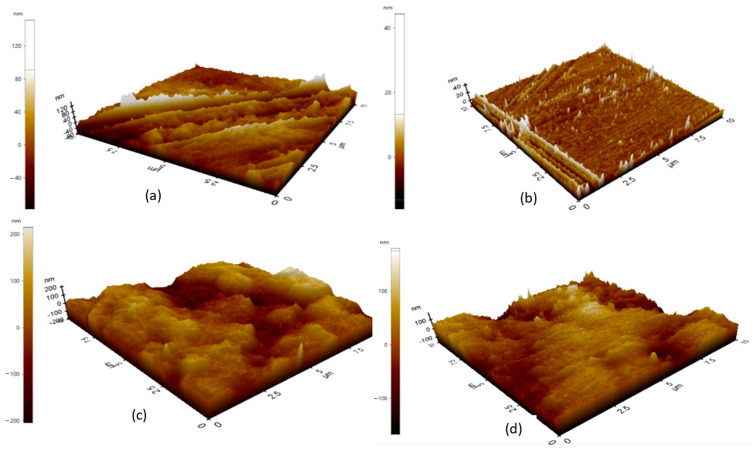
Topography maps of HDPE (**a**), LDPE (**b**), PMMA (**c**), and TPU (**d**).

The generation of triboelectric charges is influenced by surface topography, including controlling the effective contact area, the positions of the sites of charge trapping, and the enhancement of an electric field at the dielectric interface. Both forward and backward passes were taken in order to subtract artefacts due to the convolution of the tip, thermal drift and hysteresis of the scanner itself [34]. The results of the topography imaging of each of the four materials, HDPE, LDPE, PMMA and TPU, are shown in Figure 5a–d.

RMS roughness (Rq) provides a statistical statement that indicates how far the surface has eroded from the mean height of the sample material through height variability, thus giving a general idea of the surfaces or material’s total roughness or of the material’s roughness when comparing between materials. In triboelectric systems, as the RMS roughness increases, the effective contact area also increases, and therefore the amount of charge transferred during contact electrification will also be increased.

(6) $$R_{q}=\sqrt{1/L\;\int_{0}^{L}z^{2}\;(x)dx}$$

To further capture surface complexity, the developed interfacial area ratio (S_dr_) was extracted from the 3D height maps. S_dr_ quantifies the percentage increase in real surface area relative to a perfectly flat reference surface.

Beyond scalar roughness parameters, height distribution histograms were extracted from AFM height maps to assess surface uniformity. A narrow, symmetric height distribution indicates a uniform surface with consistent triboelectric behaviour, while a broader or skewed distribution suggests the presence of localised asperities or defects that may act as charge trapping sites.

The surface roughness characteristics of the polymers are represented in Table 2. Specifically, the TPU polymer has a height range from −166.987 nm to 179.604 nm, with an Rpv of 346.592 nm. This indicates that this polymer has large surface asperities. The LDPE polymer has a height range from −99.907 nm to 185.369 nm, with an Rpv of 285.277 nm, whereas this polymer has some surface undulation. The HDPE polymer has the narrowest range of heights at −87.248 nm to 161.485 nm, with an Rpv of 238.733 nm, indicating it has a more compact surface morphology than either TPU or LDPE. The PMMA polymer exhibited the largest height range of −204.435 nm to 215.232 nm, with an Rpv of 310.825 nm, indicating that there are large differences in the heights of the surface asperities of the PMMA polymer compared to other tested polymers.

Surface smoothness is determined by average roughness (Ra) and root mean square roughness (Rq). LDPE has an average roughness (Ra) of 17.330 nm and a root mean square roughness (Rq) of 22.885 nm, which indicates that it is fairly smooth. The average roughness (Ra) value for HDPE is 17.621 nm and the root mean square roughness (Rq) value equals 23.184 nm, further validating the similarity of morphology between these two polyethene-based materials. In contrast, TPU has much greater roughness values (Ra = 35.577 nm, Rq =44.592 nm), indicating a fully textured surface with many more asperities. On the other hand, PMMA has an average roughness (Ra) of 30.820 nm and a root mean square roughness (Rq) of 55.282 nm, indicating significant levels of roughness, although, the average roughness (Ra) of PMMA is lower than TPU, while the root mean square roughness (Rq) of PMMA is high because of a larger number of height variances. The 10-point mean roughness (Rz) typically indicates the strength of surface features and is derived by averaging together the five tallest peaks and five lowest valleys on the surface. The 10-point mean roughness (Rz) of LDPE is 248.809 nm, which indicates the existence of significant isolated high asperities despite having a moderate average roughness (Ra). The 10-point mean roughness (Rz) of HDPE, 230.289 nm, is slightly lower than the 10-point mean roughness of LDPE, and therefore the surface features are less pronounced than those found on HDPE.

TPU exhibits the greatest Rz value; at 324.197 nm, this is consistent with the higher values for Ra and Rq (both Rz measurements suggest and confirm very prominent surface textures). PMMA has an Rz value of 292.168 nm, indicating that PMMA experiences significant vertical variation in its surface with a wide range of asperity heights. Although the average surface roughness varies across the materials, all materials exhibit localised surface characteristics that can produce differences in triboelectric charge generation. Rsk analyses indicate that there are differences in the symmetry of peak-height distributions on the various surfaces. LDPE has Rsk = −0.154, indicating that the height distribution is nearly symmetric; this means there are relatively equal numbers of peaks and valleys, and this will produce uniformly distributed contacts when the LDPE is repeatedly cycled through contact separation. Therefore, LDPE has the most stable triboelectric output. Conversely, HDPE exhibits a more negative Rsk value (Rsk = −0.539), indicating that there is a greater proportion of valley surfaces in this material; this morphology has several recessed areas that can trap and retain charge. TPU (Rsk = 0.464) and PMMA (Rsk = 0.252) both exhibit positive Rsk values, indicating that they have an excess of peaks compared to valleys on the surfaces. Therefore, due to the nature of their surfaces, TPU and PMMA are more likely to create greater localised electric field strengths at the tips of their asperities during contact electrification, which will increase the probability that charges will transfer during contact.

### 3.2. COMSOL Multi-Physics Simulation Outputs and Performance Analysis

**Figure 6:**
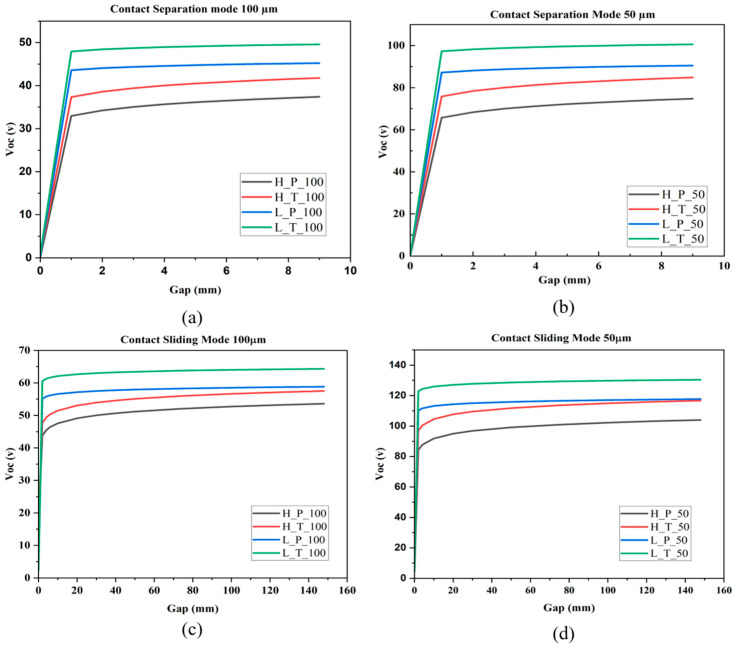
Open circuit voltage (V_oc_) of contact separation and sliding modes with 100 and 50 µm thickness. (**a**) Open circuit voltage (V_oc_) of Contact separation mode TENG at 100 μm dielectric layer thickness, (**b**) Open circuit voltage (V_oc_) of Contact separation mode TENG at 50 μm dielectric layer thickness, (**c**) Open circuit voltage (V_oc_) of Contact sliding mode TENG at 100 μm dielectric layer thickness, (**d**) Open circuit voltage (V_oc_) of Contact separation mode TENG at 50 μm dielectric layer thickness.

This electrical output characteristics of the triboelectric nanogenerator (TENG) were examined using contact separation (CS) and contact sliding (CSL) operating modes. Evaluation also considered two dielectric thicknesses (50 µm and 100 µm) for each configuration of triboelectric materials (HDPE-PMMA, LDPE-PMMA, LDPE-TPU and HDPE-TPU). Short circuit transferred charge (Qsc) and open circuit voltage (Voc) evaluations were performed to provide a basis for deriving the output performance of TENG. The results of Qsc were similar for both methodologies with respect to dielectric thickness of 50 microns, as exhibited by a maximum Qsc value of approximately 3.48 pC for the TPU (HDPE-TPU and LDPE-TPU) materials; however, the PMMA systems (HDPE-PMMA and LDPE-PMMA) both produced lesser amounts of transferred charge (approximately 3.16 pC). Regardless of the dielectric thickness increase from 50 to 100 microns, the relative difference in transferred charge between TPU and PMMA remained constant throughout the separation cycle. In addition, after completion of charge transfer, the value of Qsc remained constant as the separation distance was increased. When the dielectric thickness increased from 50 to 100 microns, the value of Qsc for all four configurations was approximately 1.75 pC with a consistent relative ranking between all four systems. The results indicate that the dielectric thickness directly affects the magnitude of transferred charge, while the triboelectric pairing determines the relative charge levels. The open circuit voltage in CS mode increases monotonically with separation gap and approaches saturation at larger distances. For the 50 µm dielectric configuration, the voltage increases rapidly at a small separation gap and stabilises beyond approximately 5 mm. The maximum Voc obtained is approximately 100 V, with the LDPE-TPU (L_T_50) system exhibiting the highest voltage, followed by LDPE-PMMA (L_P_50), HDPE-TPU (H_T_50) and HDPE-PMMA (H_P_50). For the 100 µm dielectric thickness, the maximum open circuit voltage decreases to approximately 50 V across all material combinations. The voltage gap relationship retains the same functional form as observed for the 50 µm configuration, differing only in amplitude. The consistent reduction in voltage reflects the increased effective capacitance associated with thicker dielectric layers. No change in ordering among the material combinations is observed in Figure 6a–d.

In the CSL mode, the open circuit voltage increases continuously with sliding distance for both dielectric thicknesses; for the 50 µm configuration, the voltage exceeds 130 V, with the LDPE-TPU (L_T_50) system producing the highest output. Unlike CS mode, the CSL mode does not exhibit early voltage saturation within the simulated sliding range. For 100 µm, the voltage output is reduced relative to the 50 µm case but maintains the same monotonic dependence on sliding distance. The voltage enhancement observed in CSL mode relative to CS mode is consistent across all material combinations and thicknesses.

**Figure 7:**
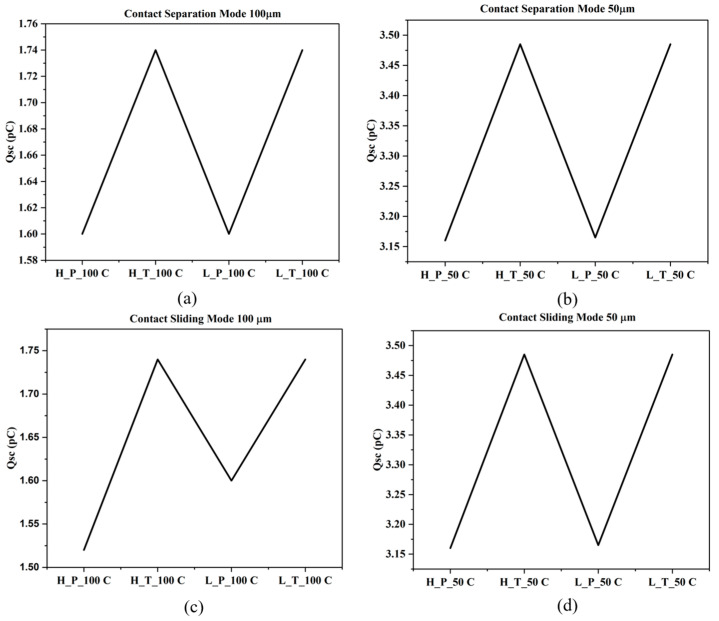
Short circuit transfer charge (Qsc) of contact separation and sliding modes with 100 and 50 µm thickness. (**a**) Short circuit transfer charge (Q_sc_) of Contact separation mode TENG at 100 μm dielectric layer thickness, (**b**) Short circuit transfer charge (Q_sc_) of contact separation mode TENG at 50 μm dielectric layer thickness, (**c**) Short circuit transfer charge (Q_sc_) of Contact sliding mode TENG at 100 μm, (**d**) Short circuit transfer charge (Q_sc_) of Contact sliding mode TENG at 50 μm.

The transferred charge in CSL mode exhibits magnitudes comparable to those obtained in CS mode for corresponding dielectric thicknesses. For 50 µm thickness dielectric, the maximum Qsc is approximately 3.48 pC, while, for 100 µm thick dielectrics, it decreases to approximately 1.74 pC. The transferred charge reaches saturation once sufficient sliding displacement is achieved, and no further increase is observed with continued motion. As depicted in Figure 7a–d the similarity in transferred charge value between CS and CSL mode for identical material and thickness parameters indicates the total charge generated per cycle is independent of the mechanical excitation mode.

### 3.3. cVAE Model Performance

#### 3.3.1. Reconstruction Accuracy

**Figure 8:**
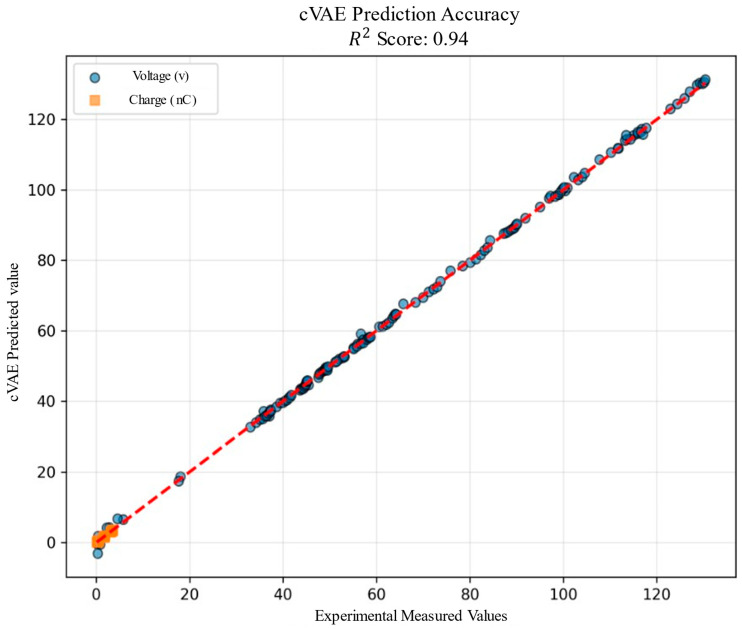
cVAE prediction accuracy plot.

The trained cVAE model achieves a parity plot comprising experimentally measured TENG output against model-predicted values, as shown in the Figure 8. The R^2^ score ≥ 0.94 on the training dataset, as confirmed by the parity plot of true versus reconstructed Voc and Qsc values. The results confirm that the physics informed feature representation comprising interfacial work function difference, combined surface roughness parameters, KPFM surface potential, calculated surface charge density computed via parallel plate capacitor model, dielectric layer thickness, gap distance, and operational mode collectively predicted the open circuit voltage and short circuit charge, exhibiting exceptionally tight agreement with experimental measurements across the full dynamic range, with data points clustering along the correspondence line with negligible systematic deviation. Along with that, short circuit charge predictions are concentrated in the low-magnitude region, consistent with the physical characteristics of the tested TENG configurations, wherein charge magnitudes are subsequently smaller relative to the voltage response. The model maintains high fidelity for both outputs simultaneously through a shared two-dimensional decoder output layer, validating the multitarget learning architecture. The near unity R^2^ score in conjunction with the probabilistic generative architecture validates that the 4-dimensional latent space successfully encodes a physically meaningful and compact representation of TENG operating manifold.

#### 3.3.2. Feature Sensitivity Analysis

**Figure 9:**
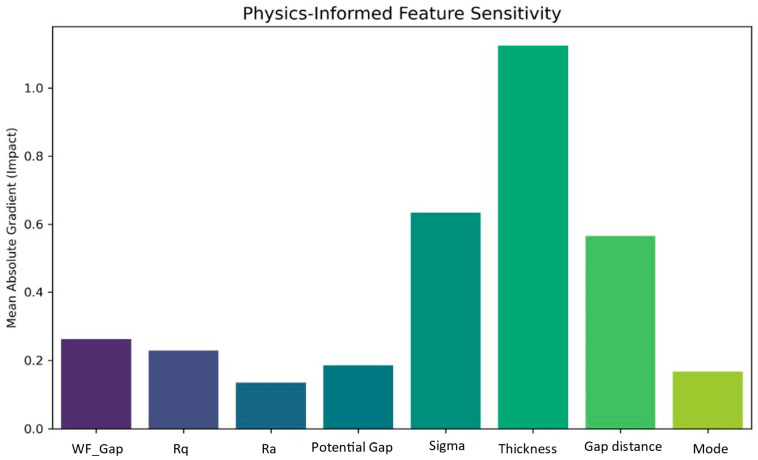
Physics-informed feature sensitivity plot.

To identify the relative influence of each physics-derived input feature on TENG electrical output prediction, a gradient-based sensitivity analysis was performed on the trained cVAE decoder network presented in Figure 9. The analysis unambiguously identifies the dielectric layer thickness as the most influential parameter governing TENG output. Along with that, gap and surface charge density are the next most influential features. This result is consistent with the parallel plate capacitor model underlying triboelectric energy generation, where the output voltage scales inversely with the dielectric layer thickness. Collectively, sensitivity analysis establishes a clear design priority framework for TENG output optimisation.

#### 3.3.3. Training Convergence

**Figure 10:**
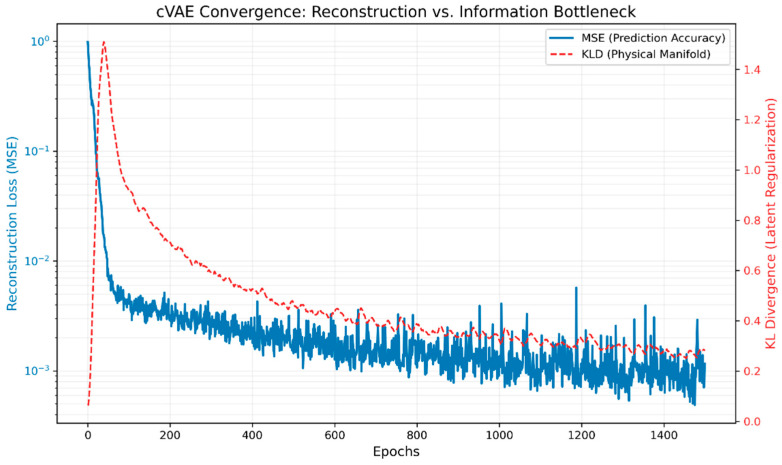
cVAE training convergence.

The convergence of the training performance of cVAE is indicated in Figure 10. The initial rapid descent of the mean squared error (MSE) loss from approximately 100 to between 10 and 12 (around the first 50 epochs) suggests that the architecture for both the encoder and decoder is learning the underlying linear relationship between a physics-informed input feature and the associated TENG electrical output. The KLD trajectory demonstrates a distinctively different convergence profile (with physical interpretation). In the first 10 to 20 epochs, there is a significant spike indicating that the model has produced an initial encoding of the maximum amount of information about the TENG experimental data into the latent space prior to the alignment by regularisation and constraints to the posterior of the output distribution. After reaching that first peak, the KLD undergoes a monotonic decay, which indicates that the subsequent process of progressively enforcing the isotropic Gaussian prior of N(0,1) across the four-dimensional latent space therefore leads to the encoder organising the physical representation from the learning process into a compact and continuously transferable manifold. After approximately epoch 800–900, both MSE and KLD losses have stabilised, which indicates that the model has achieved a consistent and well-formed evidence lower bound.

#### 3.3.4. Material-Performance Correlation Matrix Analysis

**Figure 11:**
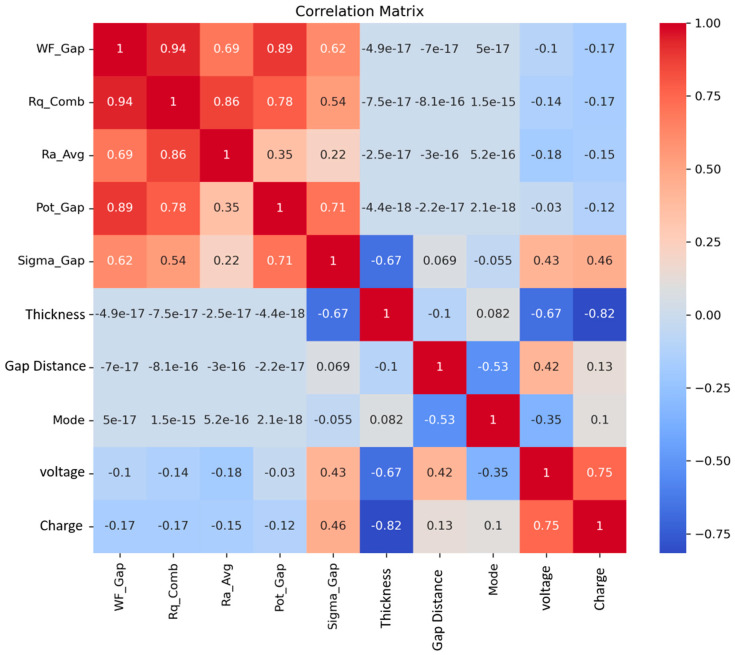
Correlation matrix for the experimental dataset.

The systematic quantification of linear independence between physics-derived input features and TENG electrical output parameters, through the correlation matrix, was computed across the experimental dataset and is presented in Figure 11. In the upper-left quadrant of the matrix, we can see a group of AFM-derived material characterisation features that have strong positive correlations with each other. Specifically, the work function gap (WF_Gap) shows very strong correlations with combined RMS roughness (Rq_Comb) and KPFM surface potential difference (Pot_Gap). This suggests that materials that have larger differences in their electronic work functions also tend to show higher degrees of surface roughness anisotropy and higher degrees of surface potential difference. The surface charge density difference (Sigma_Gap) has moderate positive correlations with WF_Gap and Pot_Gap, consistent with Sigma_Gap being derived from KPFM potential measurements using the parallel plate model. Additionally, Sigma_Gap is strongly negatively correlated with thickness and thus reflects the inverse dependence of charge density σ = ε_0_ε_r_V/d on thickness; thinner films will have larger calculated charge densities for equal surface potentials than thicker films will have. The last two rows of the matrix indicate that there are direct relationships between the input features and the electrical outputs from TENGs. Dielectric thickness exhibits the strongest individual correlation with both voltage and charge output, with the negative sign confirming the inverse thickness–output relationship predicted by triboelectric theory. Sigma_Gap shows meaningful positive correlations with both voltage and charge, confirming that the calculated surface charge density difference is a physically relevant predictor of electrical output. Sigma_Gap shows meaningful positive correlations with both voltage and charge, confirming that the calculated surface charge density difference is a physically relevant predictor of electrical output. Operational mode shows a moderate negative correlation with voltage, indicating that sliding mode tends to produce higher voltage outputs than separation mode on average across the dataset.

### 3.4. Design Space Exploration and Optimisation

#### 3.4.1. Interaction Maps: Thickness vs. Gap

**Figure 12:**
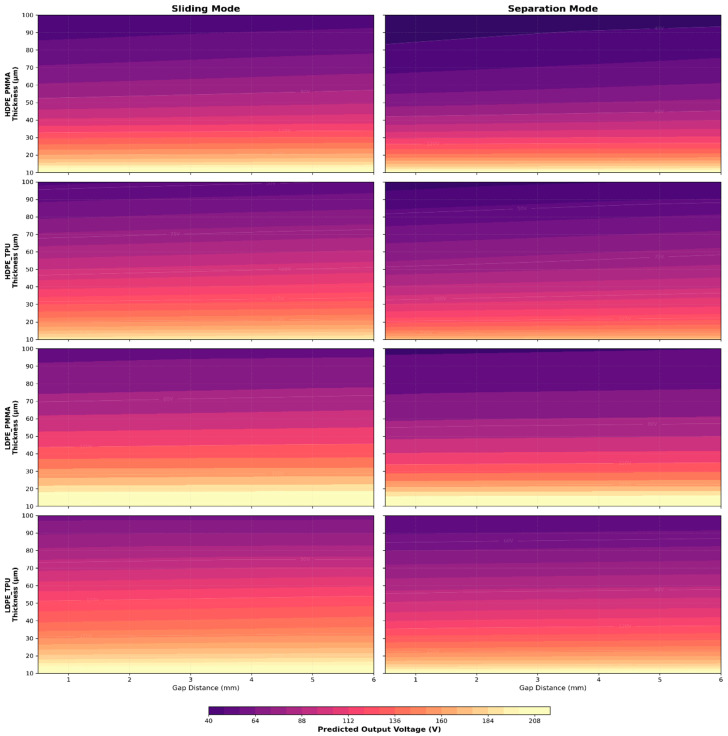
Interaction plots for predicted voltage in separation and sliding mode for various material combinations (Voc).

Using the trained cVAE, we generated 3D contour maps of predicted Voc as a function of gap distance (0.5–6 mm) and dielectric thickness (10–100 µm) for all four material pairs in both operating modes, as presented in Figure 12. As the material thickness from 100 μm to 10 μm goes down, the predicted output voltage from the interaction maps shows a dramatic increase in the electrical output voltage with significantly higher predicted output voltages in both modes for HDPE/PMMA compared to all other combinations very close to or exceeding 200 V for both modes at the minimum thickness due to higher interfacial work function differences and very high KPFM surface potential differences exhibiting similar behaviours. HDPE/TPU illustrates the greatest degree of separation mode versus sliding mode suppression by being much more affected by increases in thickness, particularly as illustrated by the much darker area of purple in the higher thickness range. Comparing across the left and right columns indicates that, in each of the four pairs of materials, the sliding mode produced consistently greater peak output voltages than the separation mode at the equivalent thickness-gap coordinates, with this effect being most pronounced at low thickness values. Additionally, the separation mode panels exhibit greater steepness in voltage gradient for thicknesses of 60–100 μm than the sliding mode panels, which implies that the separation mode degrades more significantly with increasing thickness than the sliding mode.

#### 3.4.2. Mode Comparison

**Figure 13:**
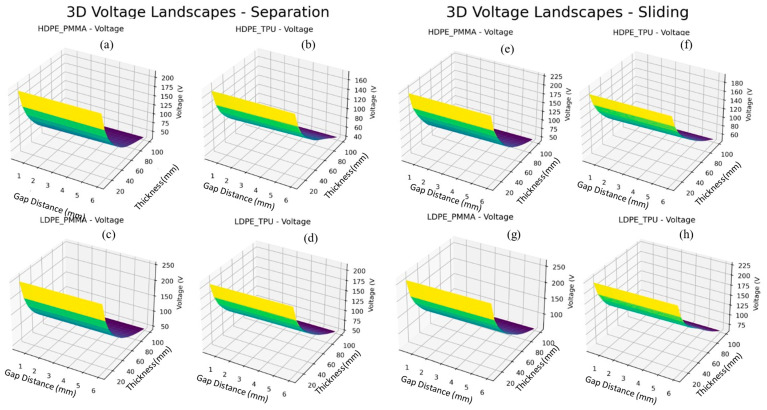
Mode comparison for predicted voltage for various material combinations for contact separation and sliding modes. (**a**) Predicted voltage landscapes for Contact separation mode TENG for HDPE_PMMA materials combination, (**b**) Predicted voltage landscapes for Contact separation mode TENG for HDPE_TPU materials combination, (**c**) Predicted voltage landscapes for Contact separation mode TENG for LDPE_PMMA materials combination, (**d**) Predicted voltage landscapes for Contact separation mode TENG for LDPE_TPU materials combination, (**e**) Predicted voltage landscapes for Contact sliding mode TENG for HDPE_PMMA materials combination, (**f**) Predicted voltage landscapes for Contact sliding mode TENG for HDPE_TPU materials combination, (**g**) Predicted voltage landscapes for Contact sliding mode TENG for LDPE_PMMA materials combination, (**h**) Predicted voltage landscapes for Contact sliding mode TENG for LDPE_TPU materials combination.

**Figure 14:**
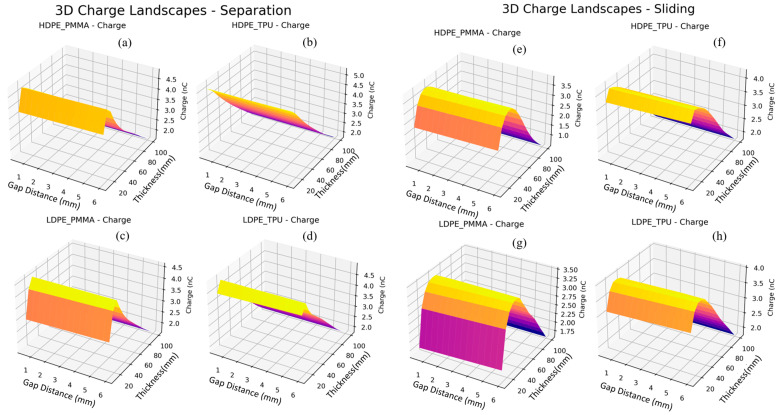
Mode comparison for predicted charge for various material combinations for contact separation and sliding modes. (**a**) Predicted charge landscapes for Contact separation mode TENG for HDPE_PMMA materials combination, (**b**) Predicted charge landscapes for Contact separation mode TENG for HDPE_TPU materials combination, (**c**) Predicted charge landscapes for Contact separation mode TENG for LDPE_PMMA materials combination, (**d**) Predicted charge landscapes for Contact separation mode TENG for LDPE_TPU materials combination, (**e**) Predicted charge landscapes for Contact sliding mode TENG for HDPE_PMMA materials combination, (**f**) Predicted charge landscapes for Contact sliding mode TENG for HDPE_TPU materials combination, (**g**) Predicted charge landscapes for Contact sliding mode TENG for LDPE_PMMA materials combination, (**h**) Predicted charge landscapes for Contact sliding mode TENG for LDPE_TPU materials combination.

Figure 13a–d and Figure 14a–d show how all material pairs and thicknesses compare systematically using a trained cVAE decoder. The predicted output voltage quickly rises as dielectric thickness decreases from 100 μm down to 20 μm, creating a well-defined high plateau at the lowest thickness for each of the two operational modes, illustrating the inverse thickness dependence, V ∝ 1/d, of the triboelectric voltage generation mechanism. The universal appearance of this ridge geometry across each panel demonstrates that thickness is the primary geometric variable controlling voltage optimisation, which corroborates the feature sensitivity analysis. Among the material pairs, LDPE/PMMA achieves the highest predicted voltage peaks in both separation (~250 V) and sliding (~250 V) modes at minimum thickness, followed closely by HDPE/PMMA (~200–225 V), LDPE/TPU (~200–225 V), and HDPE/TPU (~160–180 V). A particularly notable feature is the pronounced gap-dependent slope observable in sliding mode charge panels, where transferred charge exhibits a clear monotonic decrease as gap distance increases from 1 to 6 mm, which is most visible in LDPE/PMMA and LDPE/TPU sliding mode panels, where a sharp surface cliff is present at larger gap values. Among material pairs, HDPE/TPU achieves the highest charge output in separation mode (~5.0 nC), while LDPE/PMMA and LDPE/TPU reach comparable maxima of ~4.5 nC.

#### 3.4.3. Optimal Design Identification

Table 3 summarises the optimal predicted parameters for each mode through exhaustive cVAE. The table presents the optimum gap distance (mm), optimum dielectric film thickness (μm), predicted peak output voltage (V), and predicted transferred charge (nC). The clear finding demonstrates that the electrostatic induction efficiencies (that are experimentally validated) continue linearly with an increasing distance between the electrodes (in the 0.5–6 mm separation distance range). During the entire tested range, there is no energy-releasing gap threshold (convergence). This will physically result in an increase in the open circuit voltage with an increase in the distance apart from the electrodes due to the increasing amount of electrostatic fringe field development between triboelectric surfaces. Opposite to the common convergence distance of the gap, therefore, the optimum dielectric film thicknesses show a difference between the material pairs of both hybrid materials and thus there are two different optimum thickness regions. The TPU pairings of HDPE/TPU and LDPE/TPU have an optimum thickness range of 10.0 to 11.84 μm for both modes of operation. The PMMA pairings of HDPE/PMMA and LDPE/PMMA have an optimum thickness in the range of 11.84 to 17.35 μm, which would be slightly higher than the TPU materials. The highest voltage produced in the sliding mode of the LDPE/TPU combination was 214.54 volts with a moderate charge amount of approximately 3.49 nC. The highest sliding mode generated for the HDPE/TPU pair was approximately 4.09 nC.

## 4. Conclusions

We present a framework that integrates three distinct methodologies for predicting the electrical properties (TENG) of materials that are governed by physics and machine learning techniques: KPFM/AFM characterisation of materials, COMSOL finite element simulation, and conditional variational autoencoder (cVAE) modelling. The key outcomes and contributions of this work can be summarised as follows:

1. KPFM data collected via the parallel plate capacitor method provides COMSOL boundary condition data that is consistent with physics. This allows the use of COMSOL to model the electrical properties of TENG, as a function of material and thickness, quantitatively.
2. Theoretical scaling trends in COMSOL confirmed the expected result of halving the dielectric thickness from 100 µm to 50 µm, approximately doubling the value of Voc and Qsc. However, in both cases, using lateral sliding vs. contact separation consistently generated 25–35% higher voltages compared to contacting TENG.
3. The trained cVAE produces results with R^2^ ≥ 0.94 and provides a means to perform uncertainty-quantified, generative prediction for the entire design space. The optimal configuration identified in the study was LDPE/TPU at 50 µm in sliding mode.
4. Gradient-based sensitivity analysis provided evidence that surface charge density difference and gap distance were the primary predictors of the TENG’s electrical generation characteristics, further supporting the physical consistency of the learned model.

This framework is extensible to additional material systems and TENG geometries, and the cVAE’s generative capability can directly guide experimental synthesis and device fabrication programs. Future work will incorporate dynamic mechanical testing to account for charge decay effects, extend the model to multilayer TENG stacks, and explore transfer learning for rapid adaptation to new material families.

## Figures and Tables

**Table 1:** COMSOL simulation parameters.

	Material	Parameters
1	HDPE	Relative Permittivity = 2.30 Poisson’s Ratio = 0.46 Young’s Modulus = 0.8 Gpa Density = 950 kg/m^3^ Thermal Conductivity = 0.48 [W/(m·K)] Electrical Conductivity = 1 × 10^−15^ S/m Relative Permeability = 1 Dielectric Constant = 2.4 Surface Potential = −0.59 V Surface Charge Density = −1.25 × 10^−7^ C/m^2^
2	LDPE	Relative Permittivity = 2.25 Poisson’s Ratio = 0.49 Young’s Modulus = 0.2 Gpa Density = 920 kg/m^3^ Thermal Conductivity = 0.33 [W/(m·K)] Electrical Conductivity = 1 × 10^−15^ S/m Relative Permeability = 1 Dielectric Constant = 2.3 Surface Potential = −0.227 V Surface Charge Density = −4.62 × 10^−8^ C/m^2^
3	TPU	Relative Permittivity = 5.2 Poisson’s Ratio = 0.45 Young’s Modulus = 0.05 Gpa Density = 1200 kg/m^3^ Thermal Conductivity = 0.20 [W/(m·K)] Electrical Conductivity = 1 × 10^−11^ S/m Relative Permeability = 1 Dielectric Constant = 6.0 Surface Potential = 0.65 V Surface Charge Density = 3.48 × 10^−7^ C/m^2^
4	PMMA	Relative Permittivity = 3.0 Poisson’s Ratio = 0.37 Young’s Modulus = 3.0 Gpa Density = 1180 kg/m^3^ Thermal Conductivity = 0.21 [W/(M·K)] Electrical Conductivity = 1 × 10^−14^ S/m Relative Permeability = 1 Dielectric Constant = 3.0 Surface Potential = 1.191 V Surface Potential = 3.16 × 10^−7^ C/m^2^

**Table 2:** Surface roughness characteristics of polymers.

	Material	Ra (Average Roughness)	R_q_ (RMS)	R_z_
1	LDPE	17.330	22.885	248.809
2	HDPE	17.621	23.184	230.289
3	PMMA	30.820	55.282	292.168
4	TPU	35.577	44.592	324.197

**Table 3:** Optimal design identification.

	Material Pair	Mode	Optimum Gap (mm)	Optimum Thickness (μm)	Predicted Voltage (V)	Predicted Charge (nC)
1	HDPE/PMMA	Sliding	6	15.51	183.68	3.26
2	HDPE/TPU	Sliding	6	11.84	186.07	4.09
3	LDPE/PMMA	Sliding	6	17.35	201.26	3.01
4	LDPE/TPU	Sliding	6	11.84	214.54	3.49
5	HDPE/PMMA	Separation	6	11.84	194.6	4.3
6	HDPE/TPU	Separation	6	10	173.96	5.25
7	LDPE/PMMA	Separation	6	15.51	198.84	4.15
8	LDPE/TPU	Separation	6	10	211.47	4.56

## Data Availability

The original contributions presented in this study are included in the article. Further inquiries can be directed to the corresponding author.

## Abbreviations

The following abbreviations are used in this manuscript:

cVAE	Conditional Variational Autoencoder
ELBO	Evidence Lower Bound
HDPE	High-Density Polyethene
LDPE	Low-Density Polyethene
TPU	Thermo Plastic Polyurethane
PMMA	Poly (methyl methacrylate)
AFM	Atomic Force Microscopy
KPFM	Kelvin Probe Force Microscopy
FM-AFM	Frequency Mode Atomic Force Microscopy
TENG	Triboelectric Nanogenerators
